# Daily Evening Electronic Media Use and Sleep in Later Life

**DOI:** 10.1093/geroni/igab046.3293

**Published:** 2021-12-17

**Authors:** Yijung Kim, Shiyang Zhang, Karen Fingerman

**Affiliations:** The University of Texas at Austin, Austin, Texas, United States

## Abstract

Sleep complaints and disorders are one of the most common disturbances to health and well-being in later life. Evening electronic media use has been shown to influence the subsequent quantity and quality of sleep, but most research focused on younger age groups who are more likely to use new media (e.g., social media) to replace or complement traditional mass media such as television. To investigate how different types of evening media use is related to sleep in later life, we used ecological momentary assessment data from the Daily Experiences and Well-being Study (N = 231; Mage = 73.61) to examine how evening computer use and television viewing affect subsequent sleep hours and perceived sleep quality. Across all evening assessments, 43% of the evenings were spent using computers, and 80% of the evenings were spent watching television. Findings from a series of within-between random effects models indicated that evening computer use and television viewing had independent associations with sleep quantity and quality. That is, older adults reported fewer hours of sleep, more difficulty falling asleep, and worse overall sleep quality on nights following the evening computer use. In contrast, evening television viewing was associated with feeling less tired the next day morning. The results highlight the continued presence of television viewing in older adults’ daily lives and their distinction from general computer use. The social context in which older adults watch television in the evening may potentially explain how different electronic media use influences sleep in later life.

